# The scope and impact of mobile health clinics in the United States: a literature review

**DOI:** 10.1186/s12939-017-0671-2

**Published:** 2017-10-05

**Authors:** Stephanie W. Y. Yu, Caterina Hill, Mariesa L. Ricks, Jennifer Bennet, Nancy E. Oriol

**Affiliations:** 1 Li Ka Shing Faculty of Medicine, The University of Hong Kong, 21 Sassoon Rd, Pokfulam, Hong Kong, Special Administrative Region of China; 2 Department of Global Health and Social Medicine, Harvard Medical School, c/o The Family Van, 1542 Tremont St, Roxbury, MA 02120 USA; 3 Harvard Business School, Soldiers Field, Boston, MA 02163 USA; 4 Harvard University T.H. Chan School of Public Health, 677 Huntington Ave, Boston, MA 02115 USA; 5 The Family Van: Harvard Medical School, 1542 Tremont St, Roxbury, MA 02120 USA; 6 Harvard Medical School, 260 Longwood Ave, Suite 244, Boston, MA 02115 USA; 7 Department of Anesthesia and Critical Care, Beth Israel Deaconess Medical Center, 330 Brookline Ave, Boston, MA 02215 USA

**Keywords:** Mobile health clinics, Health disparities, Social determinants of health, Community-clinical linkage, Preventative care, Chronic disease management, Population health, Cost-effectiveness

## Abstract

As the U.S. healthcare system transforms its care delivery model to increase healthcare accessibility and improve health outcomes, it is undergoing changes in the context of ever-increasing chronic disease burdens and healthcare costs. Many illnesses disproportionately affect certain populations, due to disparities in healthcare access and social determinants of health. These disparities represent a key area to target in order to better our nation’s overall health and decrease healthcare expenditures. It is thus imperative for policymakers and health professionals to develop innovative interventions that sustainably manage chronic diseases, promote preventative health, and improve outcomes among communities disenfranchised from traditional healthcare as well as among the general population.

This article examines the available literature on Mobile Health Clinics (MHCs) and the role that they currently play in the U.S. healthcare system. Based on a search in the PubMed database and data from the online collaborative research network of mobile clinics MobileHealthMap.org, the authors evaluated 51 articles with evidence on the strengths and weaknesses of the mobile health sector in the United States. Current literature supports that MHCs are successful in reaching vulnerable populations, by delivering services directly at the curbside in communities of need and flexibly adapting their services based on the changing needs of the target community. As a link between clinical and community settings, MHCs address both medical and social determinants of health, tackling health issues on a community-wide level. Furthermore, evidence suggest that MHCs produce significant cost savings and represent a cost-effective care delivery model that improves health outcomes in underserved groups. Even though MHCs can fulfill many goals and mandates in alignment with our national priorities and have the potential to help combat some of the largest healthcare challenges of this era, there are limitations and challenges to this healthcare delivery model that must be addressed and overcome before they can be more broadly integrated into our healthcare system.

## Background

Healthcare in the United States is characterized by increasing costs and increasing chronic disease prevalence, despite continued efforts made to improve access and quality of healthcare. Moreover, the burden of certain diseases and disabilities fall disproportionately on minority groups, contributing to the health disparities seen among some in our society [1]. Without exploring and implementing new models for meeting the population’s health needs, the existing healthcare system will continue to struggle in delivering adequate and equitable health services [2].

Mobile Health Clinics (MHCs) are an innovative model of healthcare delivery that could help alleviate health disparities in vulnerable populations and individuals with chronic diseases. Indeed, some studies have concluded that MHCs are particularly impactful in the following contexts: offering urgent care, providing preventative health screenings, and initiating chronic disease managements [2]. By opening their doors directly into communities and leveraging existing community assets, MHCs can offer tailored, high-impact and affordable health care that responds dynamically to the community’s evolving needs.

Epidemiological modeling done by Mobile Health Map, a program that aims to provide a means of monitoring the characteristics and health trends of medically disenfranchised populations who visit MHCs, estimates the existence of 2000 mobile clinics nationwide. To date, approximately 36% of these clinics have registered on Mobile Health Map’s publicly available online database. Mobile Health Map encourages clinics to anonymously share aggregated demographic information about the populations they serve and the services they deliver, and the compiled data indicates that MHCs provide up to 6.5 million visits annually in the United States. These clinics offer a range of services – 42% of MHCs surveyed offer primary care, 45% offer prevention screenings, and 30% offer dental services. Many clinics also provide specialty care such as mammography, mental health monitoring, and ophthalmology checks [3].

Although some consider MHCs as “alternatives” to other healthcare models, the data reviewed in this article challenges that notion. Patients have reported that MHCs serve as a platform to help them navigate the more convoluted systems of the wider healthcare structure and to connect them with the medical and social resources in their community [4, 5]. In many contexts, MHCs can and do play an integral part in a healthcare system, providing accessible and sustainable care with quality that matches traditional healthcare settings [6–9].

## Methods

### Search method

Between January 2015 and December 2016, three reviewers (SY, CH, MR) independently conducted literature searches through the PubMed database with the search terms “Mobile Health Unit”, “Mobile Clinic” and “Mobile Health”, with and without the terms “Evaluation”, “Utilization” and “Medically Underserved Area”.

In addition to the PubMed search results, the authors also included data from and articles shared through the online collaborative research network of mobile clinics, MobileHealthMap.org. This online collaborative network was established in 2012 with funding from Health and Human Service and the Office of Minority Health and is continuing to operate under the Family Van program of Harvard Medical School. These data are self-reported and freely shared online by currently operating mobile clinic providers. The authors believe these data present a baseline of an important and emerging component of the healthcare safety-net system.

### Data extraction

All articles yielded from the literature search were reviewed for relevance using titles and abstracts. Quantitative or qualitative data that were collected within the last 20 years (since 1996), analyzed one or more mobile health clinics, focused on the mobile health sector in the United States, and provided evidence of strengths and/or weaknesses of the analyzed clinic or the mobile clinic sector were then read in their entirety. 51 articles were identified and included in the review.

One author (SY) extracted each article’s authors, publication year, methods and conclusions to be organized on an Excel sheet. Themes were then identified and compiled into the structure presented in this article. The number of articles identified for each theme and subtheme is displayed in Table 1. Additional references were included to give essential background information, and were searched also by PubMed, through MobileHealthMap.org, or in official governmental publications.

**Table 1:** Number of articles identified for each of the themes and subthemes of the review

Theme	Subtheme	Number of articles
Increasing healthcare access	Facilitating healthcare for minorities	12
	Geographical and logistical convenience	9
	Trusting provider-client relationships	6
	Emergency coverage	2
Improving health outcomes	Screenings	11
	Initiating preventative care	6
	Managing chronic diseases	3
	Enabling self-efficacy	7
Addressing social determinants of health		6
Advancing population health		3
Reducing healthcare costs	Avoidable emergency department visits	3
	Hospitalization and hospital readmission rates	1
	Symptom-free days	1
	Quality-adjusted life years	3
Mobile clinics and the healthcare reform	Private insurers	1
	Accountable care organizations	1
	Non-profit hospitals	1
Limitations of mobile health clinics	Fragmentation of care	7
	Financial issues	3
	Spatial and structural constraints	4
	Logistical challenges	1
Total		51

## Increasing healthcare access

### Barriers to healthcare access

Many studies show that Mobile Health Clinics are effective in facilitating access to health care, particularly for minority groups [6, 10–17]. Compared to the general population, minorities often have poorer health and face a higher number of barriers in accessing health services, indicating a need for healthcare agencies to reach out to these communities. According to data collected through Mobile Health Map, 52.2% of clients seen by MHCs nation-wide identify as non-White and 40% identify as Hispanic [3]. Other target populations of MHCs include vulnerable communities such as the homeless, displaced populations, immigrants, migrant workers, the under-insured, and children; historically, these groups are very often disconnected from traditional healthcare settings and require support in accessing healthcare. Even though men have been found to exhibit poorer healthcare-seeking behaviors, Mobile Health Map data highlights the ability of MHCs to attract male patients, who make up 50% of MHCs’ clients [10, 18].

Cited barriers to health care services among the general and vulnerable populations include [2, 19–23]:

- Transportation/geographic barriers
- Insurance status
- Legal status
- Financial costs
- Linguistic and cultural barriers
- Lack of healthcare providers
- Perceived absence of patient-centered care
- Psychological barriers
- Intimidation by healthcare settings
- Hours of operation
- Anonymity concerns

As outlined in the sections below, the structure, operation and staff of MHC can overcome both obvious and subtle elements of these healthcare barriers.

### Strategies of mobile health clinics

Broadly, many mobile clinics incorporate several recommendations from the Institute of Medicine’s Committee on Understanding and Eliminating Racial and Ethnic Disparities in Health Care, including (1) community health workers, (2) patient-centered care focusing on patient education and empowerment, (3) cultural competence training for staff, (4) stability and consistency of service provision within communities, and (5) staff diversity [24]. All of these elements have been shown to overcome barriers resulting from poor patient-provider communication, mistrust, and sense of disempowerment among minority communities [21, 25–29].

Of note, the Family Van clinic in Massachusetts did not see a decline in visitors in the years after the 2006 Massachusetts Healthcare Reform, and that most of Family Van’s clients (approximately 90%) are insured [3, 30]. This is consistent with evidence that there are continued barriers to primary care services apart from insurance concerns, including copayments, waiting times, complexities of navigating the system and feelings of intimidation [19, 25, 31–34]. Studies suggest that these healthcare barriers can be overcome by MHCs’ model, as discussed below.

### Geographical and logistical convenience

By delivering the necessary services right to clients’ doorsteps, often without fees and complex paperwork, many MHCs serve individuals who may not have the time, resources and motivation to travel to traditional clinics. Qualitative studies indicate that clients appreciate the convenient neighborhood locations that only mobile clinics can occupy [4, 35, 36]. MHCs embody a sense of visibility and accessibility that eliminate many logistical barriers to traditional forms of healthcare, such as transportation issues, difficulties making appointments, long waiting times and complex administrative processes, helping and encouraging vulnerable populations to receive the necessary health services [2, 37–41].

### Trusting provider-client relationships

Many successful mobile clinics cite their ability to foster trusting relationships [4, 7, 42–44]. Oftentimes, individuals become disenfranchised from their healthcare sources due to lack of trust in a system seemingly not designed for the clients’ best interest – MHCs, by their patient-centric design, are well positioned to regain the trust of these individuals and reconnect them to regular health providers. Qualitative research has found that patients value MHCs’ informal setting, familiar environment, convenient location and staff who “are easy to talk to” [25, 32, 44, 45]. Because MHCs make the effort to physically drive into communities, community members feel that the clinics are reaching out to care about them, inspiring them to take more charge of their own health [4]. Trusting relationships are further facilitated, as a communications academic argued, by MHCs’ unique use of space – these clinics’ location in familiar neighborhood areas, such as parks and shopping centers, makes the space aboard the vans an ideal blend of social and health care space, making the intimate van setting more welcoming and less intimidating [42].

### Emergency coverage

Because MHCs can be flexibly tailored to meet the needs of target communities, they can be effectively used in emergency situations when care is disrupted. For example, in January of 2016, the city of Flint, Michigan was declared to be in a state of emergency, and later a federal state of emergency, due to lead contamination in the city’s drinking water supply. Between 6000 and 12,000 children were estimated to be exposed to the contamination since 2014 [46]. A study found the blood lead levels of children younger than 5 in Flint significantly increased from 2.4% in 2013 to 4.9% in 2015, with children in disadvantaged neighborhoods experiencing the greatest elevation in blood lead levels [47]. In response to the vastly increased health needs and the low healthcare accessibility of the children affected by the water crisis, the Children’s Health Fund partnered with Hurley’s Children Clinic to bring a mobile health clinic to the area [48]. The clinic is equipped to offer multiple levels of services, from basic screenings for lead poisoning and developmental issues, to comprehensive primary care, and provides a source of medical care for children living in underserved communities of the affected area [49]. The mobility and flexibility of mobile units enable MHCs to provide the timely and necessary medical care in emergency situations.

## Improving health outcomes

### Screenings

There has been considerable national focus on safety net programs that provide community based prevention and screening, particularly for low-income and rural communities [50–52]. MHCs are shown to be effective in reaching high-risk or stigmatized populations, such as the homeless and individuals with multiple risk factors for diseases, and are able to attract different sectors of society to engage in screenings for various illnesses [16, 53–57]. For example, a study comparing a MHC in Baltimore with a comparable traditional clinic found that the percentage of clients who agreed to undergo human immunodeficiency virus (HIV) screening was higher at the MHC (54.4% in MHC vs. 7.1% in traditional clinic), and that the percentage of HIV tests that turned out positive was also higher at the MHC (5.4% in MHC vs. 2.0% in traditional clinic), indicating that MHCs facilitate more HIV screenings and are more efficient at reaching high-risk populations [58]. Because of their ability to connect with vulnerable individuals, MHCs can help identify additional cases of infectious and chronic diseases in a nontraditional setting [11–13, 59].

### Initiating preventative care

Because mobile clinics can successfully reduce barriers in access to healthcare, MHCs provide more opportunities for underserved populations to screen for various conditions and learn to properly manage their health [6, 53, 58, 60]. Researchers found that among expectant mothers living in a Miami-based minority community, clients of MHCs were significantly more likely to start receiving prenatal care services earlier compared to the other mothers accessing traditional clinics. Moreover, mothers accessing the MHCs reported significantly lower rates of pre-term and low-birth-weight infant births (4.4% vs. 8.8%), signifying the ability of MHCs to provide vital prenatal services to mothers of the minority community [61]. Hence, mobile clinics represent a potential resource to those who would not otherwise approach a health center for the necessary services and check-ups – without these services, diagnoses and treatments would be delayed, and subsequent disease management would be further complicated [43, 62].

### Managing chronic diseases

Various MHCs have demonstrated the strength of the mobile clinic model as an effective setting for chronic disease management. For example, hypertension management is notoriously difficult for patients to adhere to – nationally, only 50% of individuals diagnosed with hypertension have the condition under control, even though 80% of patients with uncontrolled blood pressure are insured [63]. In a cohort of 5900 patients who visited the Family Van between 2010 and 2012, patients who initially presented with high blood pressure exhibited average reductions of 10.7 mmHg and 6.2 mmHg, in systolic and diastolic blood pressures respectively, during their follow-up visits. These reductions are associated with a 32.2% and a 44.6% lower relative risk of myocardial infarction and stroke respectively [30]. Similarly, HABITS for Life, a MHC in New Mexico, found that its mobile clinic model is successful in improving its clients’ cholesterol status by significantly decreasing their low-density lipoprotein (LDL) levels and increasing their high-density lipoprotein (HDL) levels after 4 visits over the course of 9 months [64]. In another example, The Health Hut in Louisiana has shown that 30% of its patients initially presenting with high blood pressure at their MHC saw decreased readings over three-month periods, and a number of diabetic patients saw a decrease of 20% or more in their glycated hemoglobin (HbA1c) levels [65]. The challenge of chronic disease management is sustaining adherence to the necessary medications and lifestyle changes, and quantitative evidence from multiple MHCs signify that mobile clinics are effective in helping patients address these challenges.

### Enabling self-efficacy

Evidence indicates that MHC patients report an increased sense of self-confidence and ability to manage their chronic conditions and navigate the healthcare system [4, 14, 16, 60]. One MHC in Pittsburg revealed that the trusting relationships clients fostered on the mobile clinic motivated patients to adopt healthier behaviors [11]. Furthermore, the HABITS for Life mobile screening program noted that 78% of its screening participants engaged in healthier behavior changes as a result of having participated in the screening [64]. By bringing health care to community spaces familiar to patients, MHCs place patients in the center of the healthcare communicative process, enabling them to feel a sense of ownership, involvement and self-efficacy in the management of their conditions [14, 44].

## Addressing social determinants of health

### Disparities in social determinants of health

Despite improvements in general health outcomes and care accessibility, disparities continue to within the US healthcare system. Eliminating inequality in healthcare is a matter of much research and debate, and is one of the goals of the national health promotion campaign *Healthy People* [1]. Apart from the importance of reducing health inequities to champion social justice, there are also strong economic reasons for addressing health disparities. The 2013 Centers for and Prevention (CDC) Healthcare Disparities and Inequalities Report estimated that eliminating health disparities in 2009 would have resulted in approximately 500,000 fewer hospitalizations and saved $3.6 billion in hospitalization costs [66].

Health and access to quality healthcare are closely correlated with social factors known to be determinants of health, such as an individual’s race, socioeconomic status, living conditions and educational level [1]. According to the World Health Organization, disparities in health are mostly due to differences in social determinants of health, which affect the ability of an individual to develop a healthy lifestyle, access medical care, and ultimately control his or her health status. Often socially and economically disadvantaged, minority groups and those who live on the fringe of society are among the most vulnerable to having poor determinants of health, causing them to be adversely affected by health inequities [67]. Hence, development of innovative strategies to address not only the medical but also the social determinants of health among minority groups is imperative to improve health outcomes and reduce healthcare costs.

### The role of MHCs

MHCs are equipped to assess and respond to unmet healthcare and social needs, connecting clients to wider community resources, and successfully building capacity into healthcare systems. The merging of personal and professional discourses is postulated to help MHC staff better understand the nonmedical factors influencing their clients’ wellbeing and devise strategies to combat negative social determinants of health [42]. MHCs’ straddle between community-based and clinical settings enable them to develop the essential networks to address both the social and medical determinants of clients’ health [14]. Collaborating with local agencies such as churches, community health centers, and other hospitals and clinics, MHCs and their wide network of resources often connect community members with both medical and social services [4]. Therefore, MHCs have been cited as a viable and valuable model to help improve social determinants of health and hence health outcomes of target populations [68].

An initiative whose work highlights the strength of MHCs in addressing social determinants of health is the Outreach Van Project of the Boston University School of Medicine. The goals of this project are crafted with the understanding that both medical and non-medical factors must be addressed in order to improve the wellbeing of underserved populations in the greater Boston area. Noticing the specific needs of homeless individuals in East Boston and the lack of outreach from other agencies to this target group, organizers of this project have successfully reached out to the homeless population through their tailored services and the visual presence of their van in the community. On top of offering basic medical care appropriate to their target clients, such as blood pressure screenings and mental health services, the Outreach Van Project also seeks to improve other aspects of their clients’ lives that are pertinent to their health, by providing warm clothes, nutritious foods, and connections to homeless shelters and other resources in the area [69]. Delivering both medical and social services directly to the feet of their target population, the Outreach Van Project’s multidisciplinary approach provides a more comprehensive and sustainable solution to their communities’ health disparities, and serves as a prime example of the strength of MHCs in reaching out to and adequately supporting all the needs of their intended clients.

Some MHCs have also implemented program websites to facilitate communication among target populations and healthcare workers. Such websites help clients broaden their network of social and medical resources, provide opportunities to educate healthcare professionals on different ways to address social determinants of health, and allow mobile clinics to share knowledge and insight [64]. For example, the aforementioned Mobile Health Map, the result of a longstanding collaboration between leaders of the MHC industry, Harvard Medical School and the Mobile Health Clinics Association, serves as a networking tool for mobile health care providers to improve their MHCs’ social and medical services through increased communications and information exchange.

## Advancing population health

As the focus of our healthcare system shifts towards population health and management, MHCs are gaining increasing traction as an efficient form of healthcare delivery that improves health outcomes not only on an individual level, but also on a population one [70]. Operating in environments familiar and convenient to clients, MHCs can serve as a linkage between community-based and clinical settings, and is in a unique position to reconnect vulnerable populations with healthcare and community resources.

### CDC’s “3 buckets of prevention”

The CDC has recently developed a framework to improve population health, consisting of three areas, or “buckets”, of disease prevention:

1. Traditional clinical preventive interventions
2. Innovative preventive interventions that extend care outside the clinical setting
3. Total population or community-wide interventions [71]

MHCs fill a niche in preventative medicine as a healthcare delivery model that seamlessly integrates services from all 3 buckets. Many of MHCs’ services, such as blood pressure monitoring, are deeply rooted in traditional preventive interventions outlined in bucket 1, providing one-on-one care and consultations that are efficacious and cost effective.

As agencies offering community-specific care in a non-traditional healthcare setting, MHCs also fit into bucket 2 as a method shown to effectively connect communities with medical services for their specific needs, and provide health education extended outside of the clinic. MHCs’ visibility and entrenchment in their communities make medical and social services more accessible to their clients, offering preventative interventions outside of the traditional clinical settings.

MHCs also advance population health as an example of the strategy outlined in bucket 3. Driving into the hearts of neighborhoods to target entire populations and subpopulations of a geographic region, MHCs are able to provide target-specific interventions that extend beyond the doctor’s office. By acting as the intermediary between the population and the clinic, MHCs are in a unique position to affect health outcomes on a community-wide level.

### Community-clinical linkage

Preventative screenings and disease managements by MHCs improve the detection of chronic illness and infectious diseases among communities, especially for vulnerable populations unable to access care elsewhere. By entering communities to connect individuals to healthcare, MHCs are serving as a stepping-stone between their target community and the larger healthcare system. For example, in a Veterans Affairs-affiliated MHC, 56% of the clinic’s clients reported the MHC visit to be their first encounter and connection with the VA healthcare system [5]. Because of MHCs’ ability to segue their clients from the community to a reliable source of healthcare, the Massachusetts Partnership for Health Promotion and Chronic Disease Prevention named mobile clinics as a best practice in helping control chronic diseases and connecting community resources to clinical settings [72].

## Reducing healthcare costs

Mobile health clinics have the potential to offer a number of cost-savings benefits to the healthcare system, by prompting earlier patient care initiation, improving patients’ ability to self-manage their conditions, avoiding emergency room visits and hospital admissions, and improving the quality of life of their clients.

### Avoidable emergency department (ED) visits

Mobile Health Clinics demonstrate cost-savings by reducing unnecessary ED visits in Massachusetts as well as nationally [73, 74]. The 2015 Cost Trends Report done by the Massachusetts Health Policy Commission estimated that more than 40% of ED visits between the financial year of (FY) 2010 and FY2014 were either non-emergency or could have been managed in primary care [75]. In FY2010, the average cost per preventable/avoidable visit was USD474, and the over 1.1 million avoidable ED visits that year accrued a cost more than USD558 million [74]. Moreover, residents from communities with the lowest average incomes had more than three times the avoidable ED rate than those from communities with the highest average incomes, and rates of avoidable ED visits were higher amongst minorities compared to the general population, signifying a need for more accessible primary care to combat glaring health disparities [16, 75].

EDs represent the only source of readily available care for those who face ongoing barriers to primary care services, such as long waiting times, copayments, complexities of navigating the system and feelings of intimidation [25, 32, 33, 76–80]. Avoidable ED visit rates signify the greater health needs of the surrounding communities, and MHCs can help fill those needs by providing tailored and easily-accessible care at costs much lower than ED visits, freeing up ED resources for those who actually require emergency care and reducing total healthcare expenditure. Breathmobiles, a program that offers medical care and monitoring on mobile clinics for children living with asthma in underserved populations, analyzed 88,865 visits by 15,986 patients from November 1995 to December 2010 on 4 of their mobile clinics in Southern California, and approximated the annual cost reduction in ED visits to be at $2,541,639 [81]. Data from the Family Van estimated that visits to their MHC avoided 2851 ED visits and thus saved about $1.4 million from January 2010 to June 2012 [30]. In another analysis using aggregate data from 16 national MHCs, Mobile Health Map calculated that an approximate $561,220 is saved on avoidable ED visits per MHC per year, suggesting a total saving of over $1.1 billion per year by MHCs across the nation [3].

Hence, MHCs have the potential to avoid unnecessary ED visits and save healthcare costs.

### Hospitalization and hospital readmission rates

Care provided by MHCs has been shown to be associated with a reduction in their clients’ hospitalizations costs, which is brought about by the shorter lengths of hospitalization periods. In a study comparing traditional acute care services to mobile acute care services for the elderly, Farber and colleagues demonstrated that those who utilized traditional services averaged a hospital stay of 7.9 days costing approximately $13,187, while those who utilized mobile services averaged a shorter hospital stay of 5.8 days costing approximately $10,315 [82]. These results imply that mobile clinics are a more cost-effective method than traditional acute care services for elderly healthcare delivery.

Reductions in 7-day and 30-day readmission rates are also potential areas to explore for savings in hospitalization-related healthcare costs. In 2011, over 17% of Medicare patients and over 14% of Medicaid patients returned to the hospital within 30 days after being discharged, resulting in governmental costs of over $31 billion [83]. Medicare and other governmental efforts have imposed penalties for readmitted hospital visits, in an attempt to decrease the associated costs. At the very least, mobile health utilization has not been found to result in higher rates of readmission compared to traditional clinic utilization [82]. However, more robust indicators are needed to demonstrate the extent of MHC-related reductions in hospital readmissions.

### Symptom-free days

Monetary savings of MHCs can also be measured by the cost of symptom-free days (SFD), which incorporates costs associated with both emergency room visits and hospitalizations. Breathmobile calculated an overall increase in symptom-free days among their pediatric asthma patients, from an average of 199 SFDs at baseline to an average of 243 SFDs post-intervention, resulting in cost-savings of $79.43/day for children between 5 and 11 years old [84]. The total amount of medical costs saved outweighed the clinic’s operational costs, demonstrating a potential arena in which MHCs can contribute to lowering the nation’s overall healthcare expenditure.

### Quality-adjusted life years

Tolley and colleagues estimated that the economic value of a statistical life year, also known as a Quality-Adjusted Life Year (QALY), is $70,000 [85]. Data from the Mobile Health Map approximates that $71,714,286 in QALYs is saved per year through the collective efforts of 16 MHCs included in an analysis [3]. Individual MHCs have also shown their cost-effectiveness based on the Return-On-Investment (ROI) calculator on the Mobile Health Map website. HABITS for Life estimated that $10 million worth of QALYs were saved based on their screening efforts in the 2011 fiscal year, with a ROI of $15 dollars per dollar invested [64]. Likewise, the aforementioned 4 Southern Californian Breathmobiles estimated that $24,381,000 worth of QALYs were saved by their services within a 5-year period, with a ROI of $6.73 per dollar invested [81].

Using QALYs as a metric, MHCs’ cost savings and cost-effectiveness have been recognized in various settings.

## Mobile clinics and the health care reform

Since the healthcare amendments of recent years, different players in the healthcare system have incentives to develop new goals and emphases to adapt to the new healthcare structure. Regardless of the final reform, the flexible MHC model has the potential to fit seamlessly into a restructured health system [10].

### Private insurers

Private insurers are now responsible for the coverage of more, often sicker, individuals, and thus have an incentive to look for more innovative methods to address population health and offer preventative care in attempts to lower their overall spending. Some private insurers, such as the health insurer Highmark, have already taken advantage of the proven effectiveness of mobile clinics to reach at-risk populations who would otherwise forgo medical care until the development of full-blown diseases, at which point the cost of care would be much higher than preventative services or earlier management. MHCs emphasize preventative screenings and disease monitoring in order to maintain a higher level of general health in their clients, and hence can decrease the total healthcare costs for patients, insurers, and society [86].

### Accountable care organizations

Accountable care organizations (ACOs), a healthcare management model first described under the Affordable Care Act, are agencies clinically and financially responsible for populations of patients, and hence have motivations to both improve healthcare quality and save costs. MHCs have been shown to be a cost-saving model of care delivery that reaches multiple vulnerable populations, and would allow ACOs to flexibly identify and adapt to the changing needs of communities without having to invest in permanent infrastructure in target areas. Therefore, the mobile clinic model helps ACOs achieve their dual goal of improving health outcomes and providing cost-effective care [4].

### Non-profit hospitals

Non-profit hospitals are now expected to perform adequate needs assessments and develop appropriate strategies to address community health needs [87]. By operating directly inside the communities they serve, MHCs are well situated to fully understand the medical and social needs of community members, and have the advantage of being able to identify and provide tailored services for different populations [10]. The mobile clinic model has been shown to successfully reach and care for vulnerable populations, signifying that mobile clinics can play an important role for non-profit hospitals in at-risk communities.

## Limitations of mobile health clinics

Even though many studies have supported the unique strengths that MHCs embody, MHC workers have also pointed out potential limitations of the mobile clinic model. The limitations of MHCs described in the current literature can be separated into 4 broad categories – risk of increased fragmentation of care, issues with finances, constraints by space and clinic structure, and challenges in logistical planning.

### Fragmentation of care

Continuity of care can be difficult to maintain in MHCs, because many of these clinics are not yet fully incorporated into the healthcare system and require extensive connections with hospitals, specialty clinics, ancillary services, laboratories and pharmacies to ensure that their clients receive the appropriate level of care [2]. Many MHCs have faced problems in tracking successful patient referrals [6, 14, 88], and others have found that a substantial proportion of their patients do not attend referral appointments or cannot be followed up with [11, 53, 55]. Some MHCs have attempted strategies such as routinely calling patients to coordinate follow-up, but increased fragmentation of care remains a problem to be resolved by the MHC model [53].

### Financial issues

The cost of purchasing and maintaining a suitable vehicle is another challenge of MHCs. Often run as non-profit organizations, MHCs may not be able to secure a steady source of funding to afford the usual maintenance costs, which increase as the vehicles age [53]. A survey of mobile mammography vans found that 52% sustained financial losses from issues such as downtime from vehicle maintenance, vehicular problems, bad weather and equipment damage [89]. Another study reported that 58% of surveyed MHCs identified lack of financial capacity as their most significant obstacle [53]. Underserved communities often witness services that come and go due to shortage of funding, increasing the difficulty of MHCs to gain the initial trust of these communities [15]. Various solutions, such as cross-training of staff, corporate sponsorships, collaboration with community partners and more frequent maintenance checks, have been developed by MHCs to combat the issue of financial insecurity [53].

### Spatial and structural constraints

Because of the small area in which MHCs operate, spatial and structural constraints have been reported. Confidentiality can be difficult to maintain, since the design of mobile clinics makes it easy for clients to overhear private conversations. Disruptions of privacy are sometimes avoided by designing movable partitions within the vehicle or scheduling patients who speak different languages for the same time slot [7, 42]. Space constraints can also impact service quality – one mobile mammography unit reported that the clinic’s size only permitted the use of portable machines, resulting in a lower intrinsic quality of their imaging compared to county hospitals and leading to dissatisfaction among some of their clients [45]. Even though spatial constraints can post challenges, the tight space within which MHCs operate is also documented to contribute to a positive restructuring of patient-provider relationships [42].

Even though mobility confers unique strengths upon MHCs, it can also bring about a unique set of challenges. MHC are reliant on generators, which, if broken down, can lead to a disruption in services and cause a loss of power and temperature control. Equipment that need consistent power sources, such as refrigerators, can be difficult to support on a mobile van, and some MHCs have reported an inability to store products such as vaccinations or injectable medications due to inadequate refrigerator temperatures. Reliable Internet access, especially important for electronic medical records, can be difficult to maintain due to constant movement of the MHC [53]. These limitations must be addressed in order for MHCs to reach their full potential in serving target populations.

### Logistical challenges

The quality and quantity of services that MHCs offer can also be limited by logistical issues. Surveyors found that 33% of MHCs reported some staffing difficulties, including problems with recruitment and retention of culturally component community health workers who are experienced in collaborative efforts, comfortable with working in small spaces, and willing to accept the risks of going into underserved neighborhoods. Finding a suitable location to safely park a mobile clinic for hours at a time can also be problematic, especially in urban areas. In addition, not all communities welcome safety-net clinics, for fear that it might attract marginalized patient populations, such as the homeless or intravenous drug users, into their neighborhoods [53]. Successful implementation of MHC services depends on full engagement with and buy-in from the community throughout the planning process, and ongoing partnerships must be formed and maintained in order to ensure continued communication and collaboration of MHCs with each neighborhood.

## Future directions

While the research on MHCs is still limited, the currently available literature provides a sound baseline level of evidence that helps guide future directions in both quantitative and qualitative assessments of the scope and impact of mobile clinics. As MHCs strive to demonstrate their value to the healthcare system, a number of challenges lie ahead. With the evolving role of MHCs in the context of an ever-demanding healthcare services landscape, MHCs will need to continue developing protocols to appropriately assess and respond to the health needs of target communities. Models for improving capacity and cost-effectiveness, for example altering the service provider make-up, increasing the services offered and lowering recruitment costs, should be prioritized. In achieving economies of scale, different MHCs may consider sharing their resources and experiences as appropriate and applicable [2].

Continued research is needed to demonstrate clinics’ efficacy, both from a service quality and from a cost-effectiveness standpoint. Additional metrics, in both qualitative and quantitative domains, will need to be explored in order to maximize the benefit that MHCs can bring to various target populations and to the healthcare system as the whole. Some potential metrics include:

- Changes in clients’ health behaviors and ability to self-manage their conditions
- Clients’ perspectives on the strengths of MHCs versus traditional doctors’ clinics
- Percent and number of patients diagnosed and subsequently started on treatment for chronic conditions
- Improved clinical outcomes by specific chronic disease
- Percent effectiveness in linking underserved patients to the appropriate care or resource
- Percentage of community members who utilize MHCs’ service
- Change in prevalence of un-managed chronic illnesses in target communities

Furthermore, it is imperative to pool data from different MHCs to bolster the assessment of MHCs’ impact, improve MHCs’ credibility, and effectively disseminate significant findings. To date, many MHCs unfortunately lack the capacity to implement the necessary research; therefore, measures to improve the evaluative capacities of MHCs and demonstrate the value of mobile clinics remain a critical priority.

## Conclusions

A growing body of literature supports that MHCs are a successful and cost-effective model of healthcare delivery uniquely positioned to assess and fulfill the needs of underserved populations nation-wide. Through the act of driving directly into communities and opening their doors on the steps of their target clients, mobile clinics have been shown to be able to engage and gain the trust of vulnerable populations. Because MHCs can overcome many healthcare barriers, services provided by the MHCs have been shown to improve individual health outcomes, advance population health, and reduce healthcare costs compared to traditional clinical settings. Serving as a stepping-stone between the clinic and the community, MHCs are able to address both medical and social determinants of health, and have the potential to play an important role in our evolving healthcare system. Continuous research must be carried out to address the limitations and improve the capacity of MHCs, increase the cost-effectiveness of MHCs’ services, and mine both qualitative and quantitative data to champion a more widespread integration of MHCs into different health structures in order to combat some of the largest healthcare challenges of this era.

## Abbreviations

ACO: Accountable care organization
CDC: Centers for Disease Control and Prevention
ED: Emergency Department
FY: Financial year
HbA1c: Glycated hemoglobin
HDL: High-density lipoprotein
HIV: Human immunodeficiency virus
LDL: Low-density lipoprotein
MHC: Mobile health clinic
QALY: Quality-Adjusted Life Year
ROI: Return-On-Investment
SFD: Symptom-free days
USD: United States Dollar
